# MOCCal: A Multiomic CCS Calibrator for Traveling Wave Ion Mobility Mass Spectrometry

**DOI:** 10.1021/acs.analchem.3c04290

**Published:** 2024-01-09

**Authors:** Hannah M. Hynds, Kelly M. Hines

**Affiliations:** Department of Chemistry, University of Georgia, 302 East Campus Road, Athens, Georgia 30602, United States

## Abstract

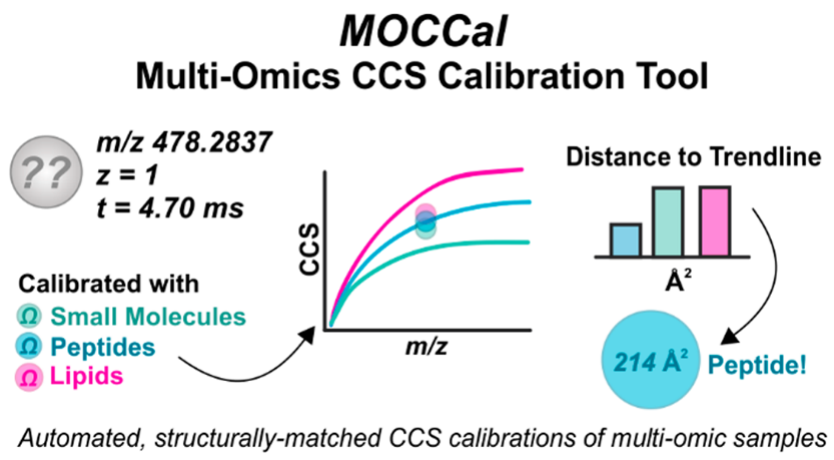

Ion mobility mass spectrometry (IM-MS) is a rapid, gas-phase separation technology that can resolve ions on the basis of their *size-to-charge* and *mass-to-charge* ratios. Since each class of biomolecule has a unique relationship between size and mass, IM-MS spectra of complex biological samples are organized into trendlines that each contain one type of biomolecule (i.e., lipid, peptide, metabolite). These trendlines can aid in the identification of unknown ions by providing a general classification, while more specific identifications require the conversion of IM arrival times to collision cross section (CCS) values to minimize instrument-to-instrument variability. However, the process of converting IM arrival times to CCS values varies between the different IM devices. Arrival times from traveling wave ion mobility (TWIM) devices must undergo a calibration process to obtain CCS values, which can impart biases if the calibrants are not structurally similar to the analytes. For multiomic mixtures, several different types of calibrants must be used to obtain the most accurate CCS values from TWIM platforms. Here we describe the development of a multiomic CCS calibration tool, MOCCal, to automate the assignment of unknown features to the power law calibration that provides the most accurate CCS value. MOCCal calibrates every experimental arrival time with up to three class-specific calibration curves and uses the difference (in Å^2^) between the calibrated ^TW^CCS_N2_ value and ^DT^CCS_N2_ vs *m*/*z* regression lines to determine the best calibration curve. Using real and simulated multiomic samples, we demonstrate that MOCCal provides accurately calibrated ^TW^CCS_N2_ values for small molecules, lipids, and peptides.

## Introduction

The use of multiple omics analysis, such as proteomics, metabolomics, and lipidomics, in combination has grown in popularity given the wealth of information that can be obtained about an organism, disease state, or condition when a systems-level view is taken.^1−3^ However, with this added sample complexity, challenges with data collection, data analysis, and feature identification also increase. The rapid gas-phase and structural separation of ion mobility mass spectrometry (IM-MS) is a promising approach for complex multiomic sample analysis because each class of biomolecule separates based on mass, charge state, and three-dimensional shape, yielding unique trends in IM-MS space.^4−8^ The addition of IM measurements provides supplementary information to the standard *mass-to-charge* (*m*/*z*) and retention time data that allows for an additional level of feature identification validation. As there are many known challenges associated with the use of ion mobility, there is always need for reliable, thorough analysis workflows that can aid in extracting the important information from the high dimensionality data.

There are many types of ion mobility devices used in modern multiomics experiments, including drift tube (DTIM), field asymmetric (FAIMS), traveling wave (TWIM), trapped (TIM), cyclic (cIM), and structures for lossless ion manipulations (SLIM).^9,10^ In classical DTIMS experiments, the amount of time it takes an ion to traverse the drift region of the spectrometer, referred to as the drift time, can be converted to a collision cross section (CCS, in Å^2^) value that represents the area of the ion-neutral collision pair. The benefit is that CCS values, unlike drift times, are comparable across different laboratories and instrumentation.^11^ Comparison of CCS values between laboratories using CCS databases can be helpful in the assignment of an identification to an unknown feature.^12−15^ However, these databases still contain only a fraction of the small molecules, lipids, peptides, proteins, etc. that are known or predicted to occur.^16^ To increase the diversity and breadth of CCS values in the absence of reference materials, several tools have been developed that use the structure and molecular descriptors of a molecule to predict its CCS value. These resources, including CCSBase,^17^ AllCCS,^15^ and CCS Predictor,^18^ increase the feasibility of using CCS values as validating evidence in unknown identifications. However, the quality of those matches relies on the precision of both experimental and predicted CCS values. Tolerances in the range of 3% are typically used to account for variability between instruments and laboratories.^16^

For DTIMS experiments, CCS values can be obtained directly from ion mobility measurements using the Mason–Schamp equation and are typically within a 2% margin between laboratories.^9,16,19−21^ However, CCS values cannot be obtained directly from the TWIM platforms. Instead, arrival times from TWIM experiments are calibrated to experimental CCS values from DTIM platforms (^DT^CCS_N2_) using a set of standards.^22^ Several different types of calibration mixtures, including poly-dl-alanine peptides, the Waters Major Mix IMS/Tof Calibration Kit, or the Agilent ESI-L Tune Mix, have been developed to provide calibration functions that cover a wide range of masses and CCS values.^22−25^ For multipass ion mobility separation techniques such as cIM and SLIM, these calibration mixtures have been advantageous in developing calibration curves based on average ion velocities rather than ion arrival times.^10^ However, it has been illustrated in previous studies that TWIM CCS (^TW^CCS_N2_) values for small molecules and lipids are most accurate, as compared to measured DTIM values, when using a calibration curve of structurally similar compounds.^7,26^ Additionally, a universal calibration mixture containing native proteins, denatured proteins, peptides, small molecules, and metabolites has been proposed for a new calibration method called IMSCal that replaces the power-law calibrations.^27^

When using TWIM platforms to study complex, multiomic mixtures, it is apparent that problems generating accurate calibrated ^TW^CCS_N2_ values can arise. Furthermore, developing high-dimensionality data analysis workflows can also pose a challenge. For this reason, we propose a novel method that can be used to assign multiomic features to their corresponding biomolecular class for ^TW^CCS_N2_ value determination without prior identification. Obtaining class assignments and accurately calibrated ^TW^CCS_N2_ values at the beginning of the data analysis process will aid the feature identification process. The proposed workflow involves the combination of CCS calibration parameter determination, biomolecular class assignment of unknown features, and the application of structurally matched CCS calibrations. These processes are encompassed within the Python program MOCCal. The accuracy of the classifications and CCS calibrations has been tested against a single-phase extract of the human serum reference material, NIST SRM 1950, analyzed using liquid-chromatography and flow-injection coupled to IM-MS. The results of the MOCCal class-specific CCS calibration approach have been compared against the recently developed IMSCal^27^ and single power law calibrations encompassing multiple chemical classes to demonstrate MOCCal’s performance for the calibration of small molecule, lipid, and peptide TWIM arrival times to ^TW^CCS_N2_ values.

## Experimental Methods

### Materials and Chemicals

Phosphatidylcholine (PC) and phosphatidylethanolamine (PE) standards (Supporting Information Document 1, Table S1) and SPLASH Lipidomix were acquired from Avanti Polar Lipids. Acetaminophen, caffeine, carnosine, *S*-(5′adenosyl)-l-methionine (SAM), and poly-dl-alanine were purchased from Sigma-Aldrich. Adenosine 5′-monophosphate (AMP) was purchased from Cayman Chemical. l-Histidine, sucrose, ammonium formate, formic acid (FA), HPLC grade acetonitrile (ACN), methanol (MeOH), 2-propanol (BuOH), and water were purchased from ThermoFisher Scientific. MassPREP enolase digest and alcohol dehydrogenase digest were purchased from Waters. NIST SRM 1950 was acquired from the National Institute of Standards and Technology (NIST).

### Standard Preparation

Calibration mixtures were prepared using lipid, small molecule, and polyalanine standards. The lipid mixture was prepared in MeOH, 0.1% FA. The polyalanine mixture was prepared in 1:1 MeOH/H_2_O, 0.1% FA. A combined lipid, small molecule, and polyalanine mixture was also prepared in 1:1 MeOH/H_2_O, 0.1% FA. The individual lipid and polyalanine mixtures along with the small molecules from the combined mixture were used for the calibration seen in this manuscript. The combined mixture as a whole was used for demonstration purposes. The final concentrations for the standards can be found in Supporting Information Document 1, Table S2. A 10× dilution of SPLASH Lipidomix was prepared by adding 10 μL of the mixture, as obtained from the vendor, to 90 μL of MeOH.

### NIST SRM 1950 Extraction

The extraction method was adapted from a previously published single-phase, multiomic method.^28^ A sample of NIST SRM 1950 was allowed to thaw on ice and was then extracted. A solvent mixture containing 60% BuOH, 20% ACN, and 20% water (BAW) was prepared. Three samples containing 3 μL of NIST SRM 1950 extract, 3 μL of 10X SPLASH Lipidomix, and 194 μL of ice-cold BAW were vortexed for 120 s and allowed to incubate at 4 °C for 1 h. The samples were then centrifuged at 3000 rpm and 4 °C for 20 min. From each aliquot, 160 μL of supernatant was transferred to 2 mL glass vials for storage at −80 °C.

### Sample Preparation

Each of the three NIST SRM 1950 extracts were prepared as a 5× dilution in BAW solvent. Additionally, 10 μL of NIST extract and 45 μL of 100 fmol/μL enolase digest were dried in a speed vacuum concentrator and reconstituted in 1:1 MeOH/H_2_O, 0.1% formic acid. The same preparation was done for 10 fmol/μL of alcohol dehydrogenase (ADH) digest and 12.7 μg/mL poly-dl-alanine as well.

### Flow Injection

Calibration solutions and NIST SRM samples spiked with peptides were analyzed by flow injection coupled to ion mobility mass spectrometry. Flow injection was performed on a Waters Acquity FTN I-Class Plus ultraperformance liquid chromatography system using a stainless-steel union and PEEK tubing (1/16 in. o.d. × 0.004 in. i.d. × 2 ft, black) in place of a column. The mobile phase consisted of 1:1 ACN/H_2_O, 0.1% formic acid (MPA, A). The flow rate was ramped from 0.075 mL/min to 0.3 mL/min over 2 min: 0–1 min, 0.075 mL/min; 1–1.4 min, ramp to 0.3 mL/min; 1.4–1.75 min, 0.3 mL/min; 1.75–2 min, ramp to 0.075 mL/min.^29^ The autosampler chamber was maintained at 6 °C. An injection volume of 5 μL was used.

### HILIC Chromatography

The diluted NIST SRM 1950 extracts were analyzed by hydrophilic interaction chromatography (HILIC) coupled to ion mobility mass spectrometry in triplicate over 3 days. The HILIC separation was performed on a Waters Acquity FTN I-Class Plus ultraperformance liquid chromatography system using a Waters AQUITY UPLC BEH amide column (2.1 mm × 100 mm, 1.7 μm) maintained at 45 °C. The mobile phases consisted of 95:5 ACN/H_2_O, 10 mM ammonium formate, 0.125% formic acid (amide A, A) and H_2_O, 10 mM ammonium formate, 0.125% formic acid (amide B, B) based on a previous method. A flow rate of 0.4 mL/min was used with the following gradient elution conditions: 0–2 min, 100% B; 2–7.7 min, ramp to 70% B; 7.7–9.5 min, ramp to 40% B; 9.5–10.25 min, ramp to 30% B; 10.25–12.75 min, ramp to 100% B; 12.75–17 min, 100% B. The autosampler chamber was maintained at 6 °C. An injection volume of 5 μL was used.

### Mass Spectrometry Analysis

Data were collected on a Waters SYNAPT XS traveling wave ion mobility mass spectrometer (TWIM-MS) in positive electrospray ionization mode with the following source conditions: capillary voltage, 3.0 kV; sampling cone voltage, 25 V; source offset, 4 V; source temperature, 150 °C; desolvation temperature, 400 °C; desolvation gas flow rate, 900 L/h; cone gas flow rate, 50 L/h. TWIM separations were performed in nitrogen with a gas flow of 90 mL/min, a wave velocity of 600 m/s (unless otherwise stated), and a wave height of 40 V. Mass calibration was performed with sodium formate over the range of 50–1200 *m*/*z*. The time-of-flight mass analyzer was operated in V-mode (resolution mode) with a resolution of ∼30 000. Data were collected with a 1 s scan time over the range 50–1200 *m*/*z*. Leucine enkephalin was used for continuous lock-mass correction during acquisition. For HILIC-IM-MS, MS/MS spectra were acquired using data-independent acquisition (MSe) with a ramped collision energy (from 15 to 45 eV) in the transfer region of the instrument.

## Results and Discussion

MOCCal utilizes biomolecule-specific TWIM CCS calibration equations based on the power law to generate calibrated CCS values for a molecular feature of unknown identity. Every singly charged feature is calibrated with as many as three different calibration equations, provided that data for small molecule, lipid, and peptide (*z* = 1) CCS calibration mixtures are provided. These calibrated CCS values are then compared against the corresponding ^DT^CCS_N2_ vs *m*/*z* biomolecular trendlines. For example, a feature calibrated with the peptide (*z* = 1) power law parameters will be evaluated against the peptide (*z* = 1) ^DT^CCS_N2_ vs *m*/*z* trendline, whereas the outcome of calibrating the same unknown feature calibrated with the lipid power law parameters will be evaluated against the lipid ^DT^CCS_N2_ vs *m*/*z* trendline. The calibrated ^TW^CCS_N2_ value with the smallest ΔCCS to the corresponding trendline is selected as the correct match, and that calibrated ^TW^CCS_N2_ value is retained. The following sections describe each step of the MOCCal workflow in detail.

### Determination of CCS Calibration Parameters

The CCS calibration process begins by selecting calibration standards for the biomolecular classes that are expected to be within the data. It is highly recommended that standards be chosen to span the desired mass range and calibration data be collected via flow injection or direct infusion. The calibrants can be mixed together into either a single solution or multiple class-specific solutions. For calibration input into MOCCal, a spreadsheet containing the calibrant names, *m*/*z* values, drift tube CCS (Å^2^) values, biomolecular class, and charge state is required. If the calibration data are processed before using MOCCal, a column containing arrival times of the calibrants should be included in the spreadsheet as well. However, if the calibration data are not already processed, raw data for each calibration solution should be loaded as a .mzML file. When raw data are loaded, arrival time data for the calibrants are extracted using the open-source software DEIMoS^30^ and fit to a Gaussian distribution. The apex of the Gaussian distribution is recorded as the feature’s arrival time. The DEIMoS or user-provided arrival times can then be used for CCS calibration. Parameters are optimized for class-specific power law regressions using the calibrant’s arrival time and reference drift tube CCS value. Users must also supply the EDC delay coefficient, which is unique to each instrument. The equations involved in these calculations can be seen in Supporting Information Document 1, eqs 1–3.

**Figure 1:**
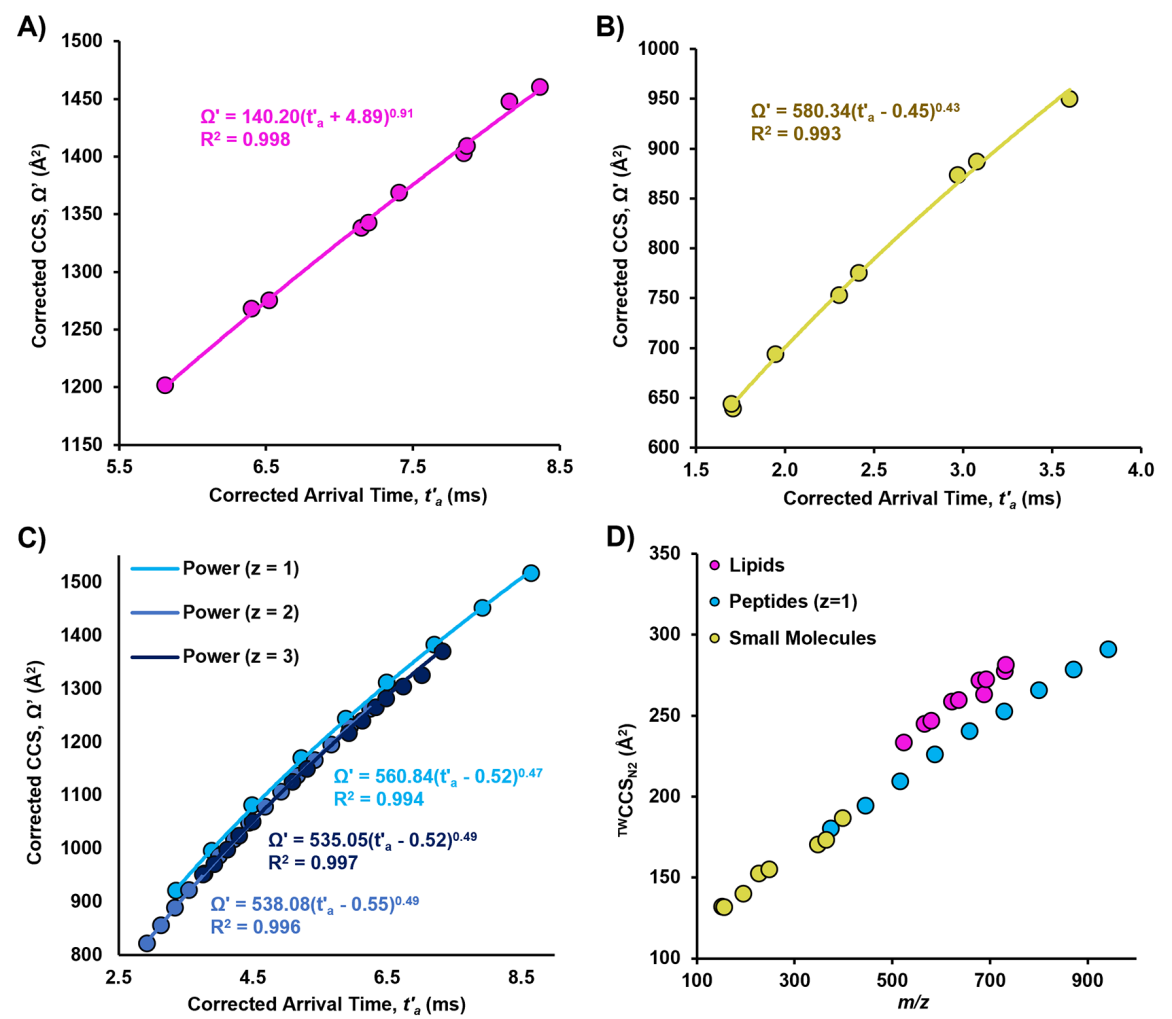
Examples of calibration curves relating calibrant arrival time (*t*′_a_, Supporting Information Document 1, eq 1) to CCS (Ω′, Supporting Information Document 1 eq 3) can be seen for (A) lipids, (B) small molecules, and (C) peptides. Three calibration curves are developed for charge states *z* = 1–3 of peptides. (D) ^TW^CCS_N2_ vs *m*/*z* values of lipid, small molecule, and peptide calibrants averaged over 3 days.

Previous work has demonstrated that power law TWIM CCS calibrations provide the most accurate results when calibrants are structurally similar to the analytes. MOCCal is currently set up to perform CCS calibrations for lipids, small molecules, and peptides (*z* = 1 to *z* = 3) in positive ionization mode. The calibration curves in Figure 1 demonstrate that each biomolecular class has distinct fit parameters for the power law calibration curve. These differences occur not only because the calibrants cover different ranges of CCS and arrival time but also because the expansion of each type of biomolecule in size and mass is influenced by the unique functional groups and structural motifs inherent to that chemical class.

### Application and Assignment of CCS Calibration Parameters to Unknown Features

Experimental data can be imported as a spreadsheet containing feature identifiers along with their corresponding *m*/*z*, charge state, retention time, and arrival time values. Based on provided charge states, features with two or more charges will be calibrated with only the peptide *z* = 2 or *z* = 3 calibration curves. For each feature in the spreadsheet with a charge state of 1, MOCCal will calculate up to three calibrated ^TW^CCS_N2_ values using each set of class-specific calibration parameters (Supporting Information Document 1, eq 4) for small molecules, lipids, and peptides (z = 1). MOCCal then uses the biomolecular ^DT^CCS_N2_ vs *m*/*z* trendlines to identify the calibration curve that provided the most accurate calibrated ^TW^CCS_N2_. A correctly calibrated feature should fall within a few Å^2^ of the ^DT^CCS_N2_ vs *m*/*z* trendline for its true biochemical class. To make this determination, the *m*/*z* of the feature is used to calculate a predicted ^DT^CCS_N2_ value based on ^DT^CCS_N2_ vs *m*/*z* regression lines developed from ^DT^CCS_N2_ values in the Unified CCS Compendium (Supporting Information Document 2, Tables 1–3).^6^ Next, the ΔCCS between the trendline ^DT^CCS_N2_ value and the calibrated ^TW^CCS_N2_ value is determined. If data for two or three types of calibrants are provided, the distance between trendline and calibrated CCS values will be determined for each biomolecular class (e.g., the lipid-calibrated ^TW^CCS_N2_ value will be evaluated against the lipid trendline ^DT^CCS_N2_ value). The most accurate calibration will be the most structurally matched to the feature’s identity and will minimize the difference between the calibrated ^TW^CCS_N2_ and trendline ^DT^CCS_N2_ values.

**Figure 2:**
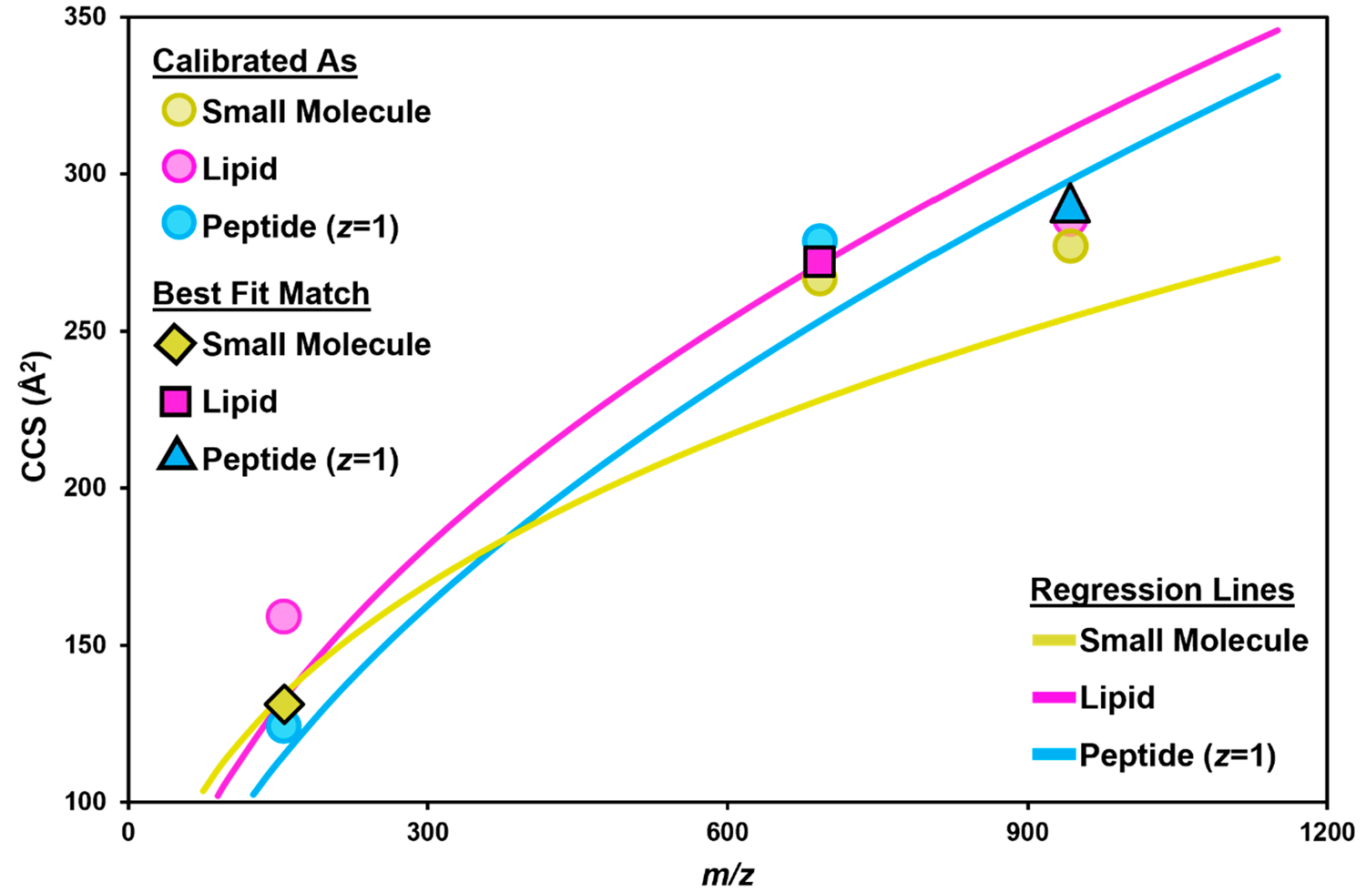
Examples of class assignment based on calibration-trendline ΔCCS calculations. Each set of shapes corresponds to a singular feature. ^TW^CCS_N2_ values are calculated for each feature using the lipid, small molecule, and singly charged peptide calibration curves. Those values are then compared to their corresponding regression lines. The class assignment is based on the smallest ΔCCS, represented by the shapes with black outlines.

The assignment process is demonstrated in Figure 2 for a small molecule, a lipid, and a singly charged peptide (see Supporting Information Document 1, Figure S1 for enlarged plots). The small molecule feature that has been calibrated with the small molecule power law equation falls directly on the small molecule ^DT^CCS_N2_ vs *m*/*z* trendline, whereas the lipid calibrated version lies well above the lipid ^DT^CCS_N2_ vs *m*/*z* trendline. The results for the lipid and peptide features are more subtle, with all calibrated values falling close together. However, it is clear that the structurally matched calibration yields a ^TW^CCS_N2_ value that is closer to the trendline matching the feature’s true identity. When calibrated with the lipid-parametrized power law equation, the feature at *m/*z 692.517 (a lipid) is 0.8 Å^2^ from the lipid trendline. The same feature calibrated with the small molecule and peptide (*z* = 1) power law equations is 38.7 Å^2^ and 25.3 Å^2^ from the small molecule and peptide (*z* = 1) trendlines, respectively. Based on these results, *m*/*z* 692.517 would be assigned as a lipid and the ^TW^CCS_N2_ value derived from the lipid-parametrized power law would be retained. Comparison of the calibrated values to ^DT^CCS_N2_ values for these features confirms that the structurally matched calibration provides the most accurate ^TW^CCS_N2_ value. For the lipid, the calibrated ^TW^CCS_N2_ value from the lipid-fit power law is 0.18% from the ^DT^CCS_N2_ value whereas the small molecule and peptide power laws yield calibrated ^TW^CCS_N2_ values with 1.8 and 2.6% error, respectively. The calibrated ^TW^CCS_N2_ value providing the smallest ΔCCS relative to its respective trendline is retained for the MOCCal output so long as it fits within reasonable *m*/*z* constraints for the class (i.e., less than *m*/*z* 550 for small molecules; greater than *m*/*z* 300 for lipids). If the feature falls outside the *m*/*z* constraints for the assigned class, the next closest match by ΔCCS will be selected.

### Scoring of Calibrated CCS Values

There are some gray areas, such as *m*/*z* 300–600, within IM-MS conformation space where multiple calibration curves can yield similar calibrated CCS values. For CCS calibrations performed with two or more singly charged biomolecular classes, every assignment is given a score from 1 to 4 to indicate the effect of the calibration on the resulting value (Supporting Information Document 1, Table S3). A score of 4 indicates that there is a difference of 6% or greater between the calibrated CCS value derived from the assigned class and the next closest match. A score of 3 corresponds to a difference between 3 and 6%. Scores of 3 or 4 illustrate that the CCS values yielded from different calibrations are substantially different from one another. However, a score of 2 corresponds to a difference between 1 and 3%, and a score of 1 has a difference of less than 1%. Scores of 1 or 2 show that at least two calibration curves from different classes yield similar results. However, the scoring process does not provide an interpretation of the accuracy of the calibrated CCS value relative to drift tube CCS measurements. A default score of 0 is given to multiply charged assignments. More details on the process of calculating the calibration effect scores and various examples with interpretation can be found in Supporting Information Document 1, sections 6 and 7.

Calibration errors of ±0.5 to ±1% are typical for TWIM CCS calibration using the power law, even in the case of structurally matched calibrants. Using the calibration-trendline ΔCCS approach, it was important to determine the effects that calibration errors may have on the assignment of an unknown feature to its correct class and calibrant set. The tolerance for CCS calibration errors within each molecular class was determined by using values from the CCS Compendium that were adjusted by ±0.5, 1.0, 2.0, and 5.0% to replicate a realistic range of calibration errors. These values were then provided to MOCCal for assignment to the biomolecular classes based on the ΔCCS method. Table 1 contains the percentage of correctly assigned features at each level of error for the three groups of biomolecules.

**Table 1:** Percentage of Features Correctly Assigned by MOCCal Using CCS Compendium Values with ±0.5 to ±5% errors

	% deviation from CCS Compendium value								
	–5%	–2%	–1%	–0.5%	0.0	+0.5%	+1%	+2%	+5%
Lipid	26%	60%	76%	84%	90%	93%	95%	97%	98%
Small molecule	81%	82%	82%	82%	82%	82%	83%	83%	81%
Peptide (*z* = 1)	92%	94%	88%	84%	79%	74%	69%	58%	21%

Despite the added errors, the small molecules from the CCS Compendium are assigned to the small molecule calibrations 81–83% with no clear influence of the positive or negative errors on the accuracy. On the other hand, increasingly negative errors applied to the CCS values of Compendium lipids lead to a drop-off in the classification accuracy. With increasing positive errors, the assignment accuracy for lipid continues to increase since there is no trendline with higher CCS values. The opposite trend is observed for the singly charged peptides, where increasing positive errors leads to a gradual decline in MOCCal’s classification accuracy and greater negative errors are increasingly assigned to the peptide calibrants because there is no trendline with lower CCS values. Based on these results, MOCCal provides the best assignment results for lipids and singly charged peptides when the calibration errors are ±0.5% but still achieves the correct assignment for ≥70% of lipids and peptides with errors of ±1%.

### Evaluation of MOCCal on Complex Multiomic Samples

We used single-phase extracts of a plasma reference material, NIST SRM 1950, to test the performance of MOCCal on a complex mixture containing metabolites and lipids with and without the addition of protein digestion reference materials. Experiments were performed on 3 days, with CCS calibration data collected each day prior to analysis of the reference material. Calibrant information can be found in Supporting Information Document 2, Table 4. Raw data files for the calibration mixtures were loaded into MOCCal as .mzML files. DEIMoS extracted the arrival times from the raw data, while MOCCal calculated the optimized parameters for class-specific calibration curves, the CCS values for each calibrant, and the calibration error relative to drift tube values. The average standard deviation of the calculated CCS values over 3 days was 0.13 Å^2^, with an average relative standard deviation (RSD) of 0.06%. The average percent errors for lipids, small molecules, and singly charged peptides were 0.04%, 0.76%, and 0.02%, respectively. Additionally, the root-mean-square error (RMSE) for lipids, small molecules, and singly charged peptides was 0.28, 0.58, and 0.12, respectively.

**Figure 3:**
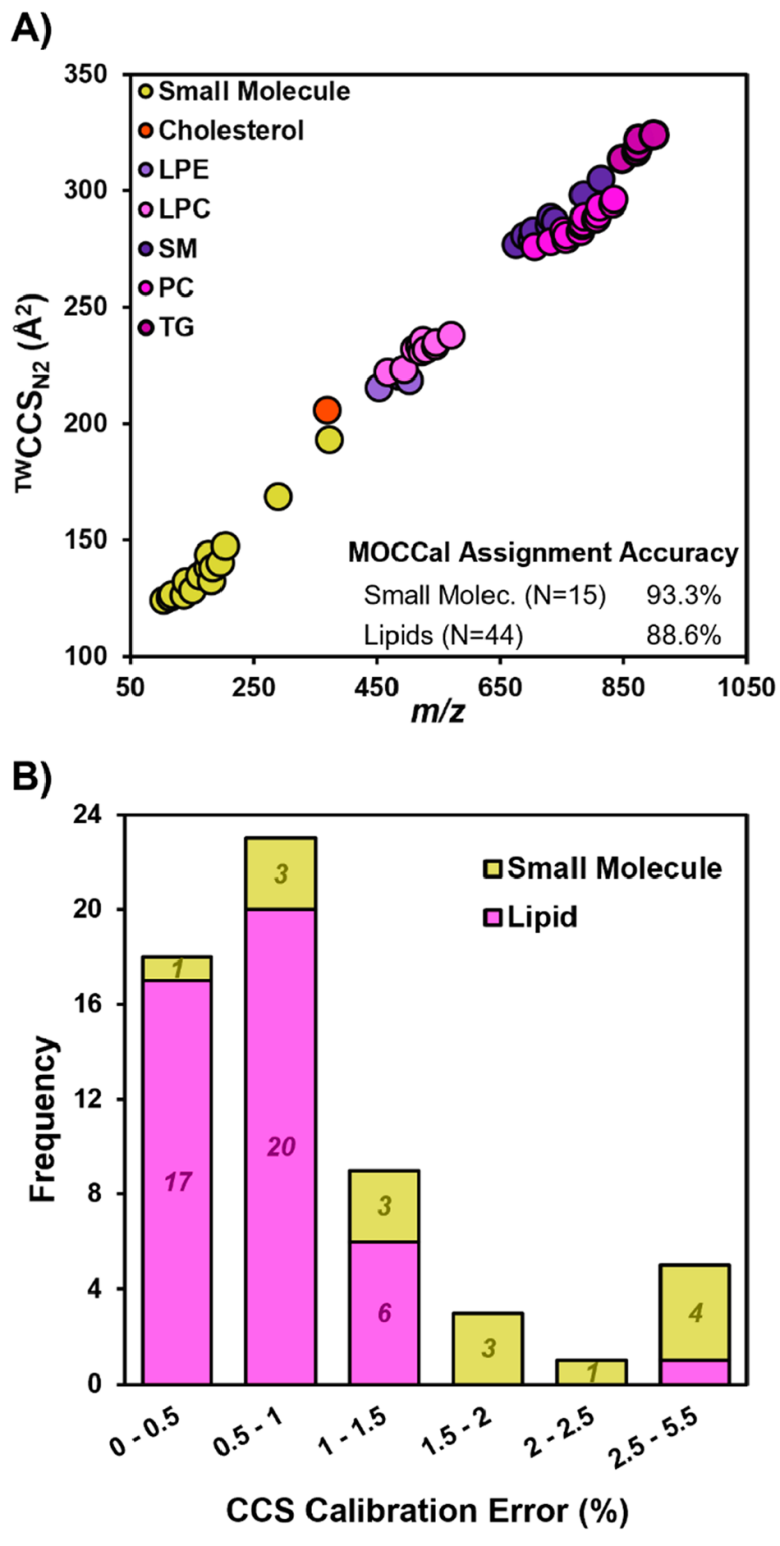
(A) IM-MS plot of calculated ^TW^CCS_N2_ values versus *m*/*z* values of NIST SRM 1950 extract lipids and small molecules averaged over 3 days, acquired using LC-IM-MS. A table with assignment accuracy has been included. (B) Histogram of NIST SRM CCS% errors averaged over 3 days.

Extracts of NIST SRM 1950 containing plasma lipids and metabolites were analyzed by HILIC-IM-MS. The data were processed in Progenesis QI (Nonlinear Dynamics/Waters Corporation) software to extract and align features based on *m*/*z* and retention time, after which arrival times for each feature are extracted. The resulting spreadsheet was loaded into MOCCal for CCS calibration. Feature identification was performed with both HMDB and an in-house Python script that evaluated the exact masses and CCS values of the features against the LipidPioneer and CCS Compendium databases.^6,31^ Consistent with previous investigations of NIST SRM-1950, several different classes of lipids and small molecules were detected in the HILIC-IM-MS data of the single-phase plasma extract.^32−35^ The metrics and calibration effect scores from the MOCCal processing of the HILIC-IM-MS data set are presented in Supporting Information Document 2, Table 6. MOCCal assigned 88.6% of the 44 lipid features to the lipid CCS calibrants, whereas 93.3% of the 15 small molecule features were assigned to the small molecule CCS calibrants (Figure 3A). Five lysophospholipids were assigned by MOCCal to the singly charged peptide calibration, which provided calibration errors of 0.5–1.8% from drift tube or predicted CCS values (values used for comparisons are listed in Supporting Information Document 2, Table 5). The peptide-calibrated lysophospholipids included sodium adducted LysoPCs, a polyunsaturated LysoPE, and the d_7_-LysoPE 18:1 internal standard. The crossover point between assignment to the lipid vs peptide calibration is demonstrated for LysoPCs in Supporting Information Document 1, Figure S2. These results demonstrate an important advantage of MOCCal for the minimization of calibration errors by implementing identity-agnostic calibration optimization for each ion.

One small molecule, trazodone (*m*/*z* 372.159), was assigned to the lipid calibration by MOCCal. Experimental CCS values for trazodone in CCS base range from 188.7 Å^2^ to 192.3 Å^2^ (1.89% difference), whereas the calibrated CCS value using the lipid power law parameters provided a CCS value of 193.0 Å^2^ (an error of 3.2% from 188.7 Å^2^ and 0.32% from 192.3 Å^2^). All lipid and metabolite features were within 5.5% of literature CCS values following calibration with MOCCal.^17^Figure 3B shows the distribution of calibration errors across the two classes. While all but one of the lipid features had errors less than 2%, a third of the small molecules had calibration errors between 2 and 5.5%. Because these small molecules all have CCS values less than 150 Å^2^, even differences of 3–7 Å^2^ from the expected values can result in a large error. For example, two of the three smallest identified small molecules yielded the two largest CCS errors of 4.8% and 5.4%, with all other errors being less than 3%. These results highlight the effect that the smallest change in CCS calibration can have on the variability of values resulting from features in the 100–300 *m*/*z* range.

A simulated multiomic mixture was created by adding poly-dl-alanine, alcohol dehydrogenase (ADH) digest, or yeast enolase digest into the NIST SRM 1950 plasma extract. Each sample was analyzed three times by flow injection-IM-MS on the same day with CCS calibration performed before experimental data collection. The MOCCal CCS calibration workflow was the same as for the LC experiments above, with the addition of doubly and triply charged polyalanine peptides as calibrants. The calibration errors for lipids, small molecules, and peptides (*z* = 1, 2, 3) were 0.02%, 0.75%, 0.01%, 0.06%, and 0.13%, respectively. The RMSE for lipids, metabolites, and peptides (*z* = 1, 2, 3) were 0.28, 0.56, 0.09. 0.09, and 0.24, respectively.

**Figure 4:**
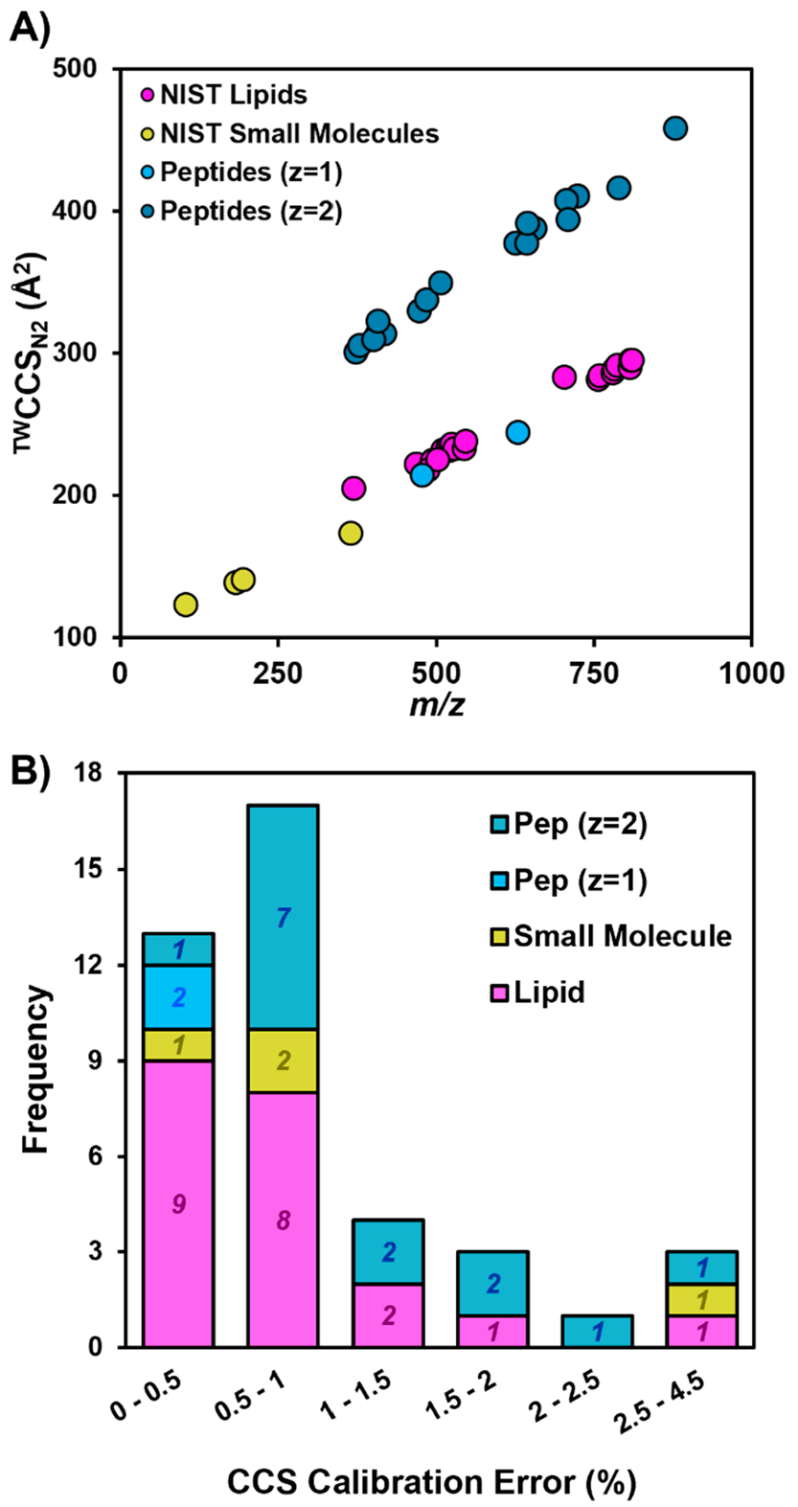
(A) IM-MS plot of class-specific calculated ^TW^CCS_N2_ values versus *m*/*z* values of NIST SRM 1950 lipids and small molecules and enolase/alcohol dehydrogenase tryptic digest singly and doubly charged peptides, acquired using FI-IM-MS. (B) Histogram of NIST SRM lipid and small molecule and enolase/ADH singly and doubly charged peptide CCS% errors. Four of the doubly charged peptides identified in the enolase digest could not be evaluated due to the absence of ^DT^CCS_N2_ values.

Similar to the LC results, one lysophospholipid in the FI-IM-MS analysis of the plasma extract (Figure 4) was assigned to the singly charged peptide calibration. The other lipids and small molecules (*n* = 24) were assigned to their structurally matched calibration and, excluding one small molecule, had calibrated CCS values within 1.7% of literature CCS values (Supporting Information Document 2, Table 7). A total of 33 polyalanine peptides were detected in the pseudomultiomic mixture, of which 6 peptides were classified as singly charged, 14 as doubly charged, and 11 as triply charged. Two small, singly-charged polyalanine peptides (Poly-Ala6, *m*/*z* 445.24; Poly-Ala7, *m*/*z* 516.28) were assigned to the small molecule calibrants but still had calibrated ^TW^CCS_N2_ values within 0.6% and 1.2% of ^DT^CCS_N2_ values. The calibrated ^TW^CCS_N2_ values for the doubly and triply charged polyalanine peptides, which are automatically assigned based on their charge states rather than the trendline fitting process, were within 0.62% of the drift tube values from the CCS Compendium. Since polyalanine was used to generate the peptide calibration curves, plasma extracts spiked with enolase digest and ADH digest were used for a more realistic test of MOCCal. A total of 20 peptides were detected between the two protein digests, including two singly charged and 18 doubly charged peptides. The calibrated ^TW^CCS_N2_ values for the ADH and enolase peptides were within 2.6% of experimental ^DT^CCS_N2_ or ^TIM^CCS_N2_ values.^19,36^Figure 4B shows the distribution of calibration errors for the lipids, small molecules, and peptides from the spiked NIST plasma. Peptide identities and MOCCal metrics for these data can be found in Supporting Information Document 2, Table 8.

When ions of different mobilities are analyzed together, as in a multiomic sample, it can be beneficial to implement a dynamic rather than static traveling wave velocity. By ramping the wave velocity, both high and low mobility ions are efficiently transported through the TWIM cell without sacrificing the separation. As with static wave velocities, the power law CCS calibration approach has proven successful for generating accurate ^TW^CCS_N2_ values when ramped TW velocities are implemented.^7,16^ We demonstrate in Supporting Information Document 1, Figure S3 that MOCCal performance for multiomic data collected under ramped wave velocities is comparable to its performance for the static wave velocity approach.

### Comparison of MOCCal against Other Calibration Approaches

A new approach for TWIM CCS calibration based on Bayesian statistics has been developed that incorporates parameters of ion motion through the TWIM cell, such as velocity relaxation and radial distribution, to eliminate the need for structurally matched CCS calibrations.^27^ This approach, as implemented in the corresponding software tool IMSCal, uses small molecules, lipids, peptides, and proteins (native and denatured) to fit a single calibration function covering a mass range of 3.5 decades. Added benefits of the Bayesian approach included improved precision and applicability to complex traveling wave conditions or multipass TWIM separations. With the ability to span several orders of magnitude of molecular mass, from native proteins to small molecules, IMSCal shows a promising new direction for CCS calibrations.

**Figure 5:**
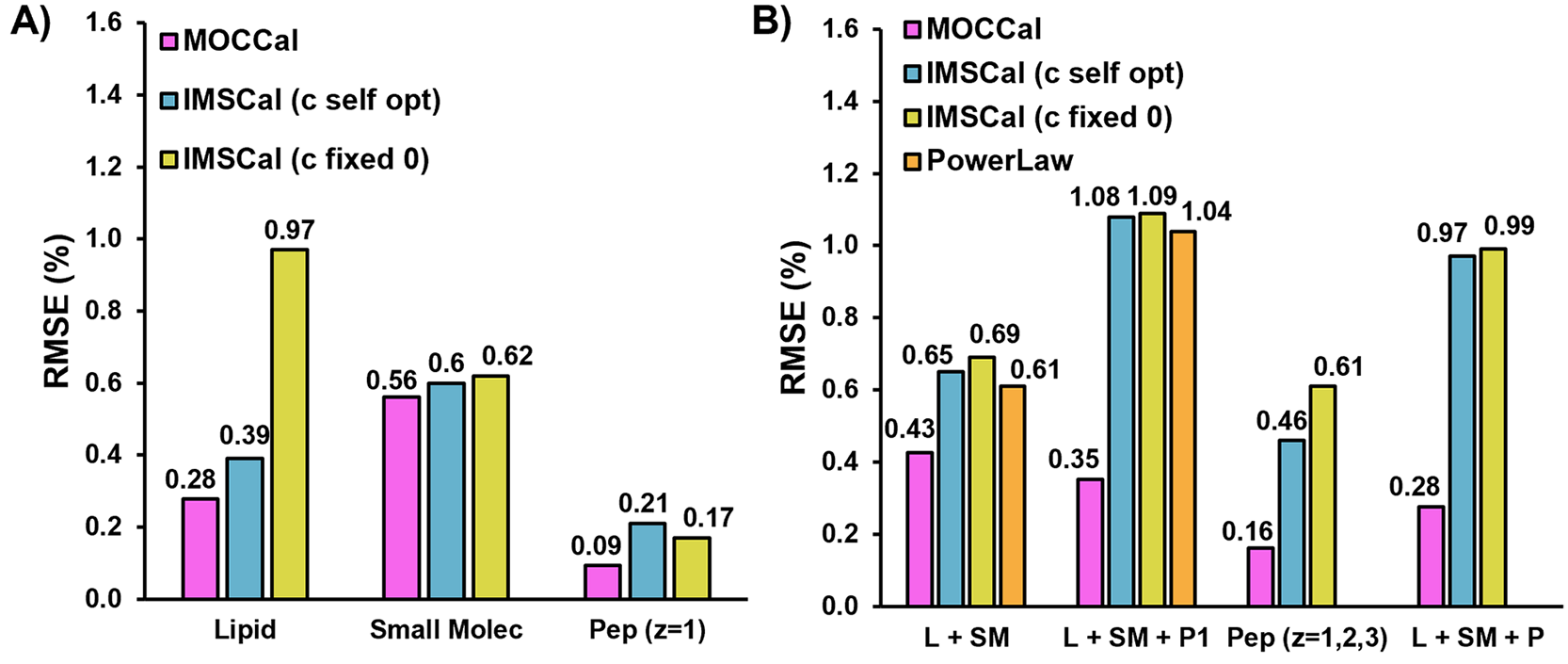
Root-mean-square error of calibration using various combinations of lipid, small molecule, and peptide calibrants. Calibration was performed with IMSCal, PowerLaw, or MOCCal. For IMSCal calculations, “*a*” was set to 1 and “*c*” was either set to 0 or was allowed to self-optimize. For PowerLaw calibration, a single power law regression built on all calibrants was used to calibrate all classes. For MOCCal, all power law calibrations were class specific. (A, left) Single class calibrations for lipids, small molecules, and singly charged peptides (B, right) Multiclass calibrations for lipids and small molecules (L + SM); lipids, small molecules, and singly charged peptides (L + SM + P1); singly, doubly, and triply charged peptides (Pep *z* = 1, 2, 3); and all calibrants (L + SM + P).

While the recommended minimal calibration set provides accurate and precise CCS values for peptides, denatured proteins, and native proteins, only three small molecules and eight lipids were included in the full, four class calibration mixture. The results of the Bayesian calibration method for these eight analytes showed minimal improvement over the traditional power law approach, where both routinely yield calibrated ^TW^CCS_N2_ values within 1% of experimental values. We evaluated how MOCCal performed against individual and mixed species calibrations using IMSCal and mixed species power law calibrations. The results of this evaluation are shown in Figure 5, and calibration parameters for all approaches can be found in Supporting Information Document 2, Table 9. For single class calibrations (Figure 5A), the calibration errors, i.e., the errors from applying the calibration equation back onto the calibrants, for MOCCal are slightly better than IMSCal for single-class calibrations. There is a noticeable performance difference for IMSCal calibration of lipids when the radial parameter, *c*, is fixed at a value of zero as suggested in the IMSCal documentation.^27^ The calibration errors for multispecies (*z* = 1) calibrations (Figure 5B) using IMSCal (both *c* = 0 and self-optimized *c* term) were comparable to multispecies (*z* = 1) power law calibrations. However, calibration errors for all multispecies approaches were greater than the single-species power law calibration method implemented in MOCCal.

**Figure 6:**
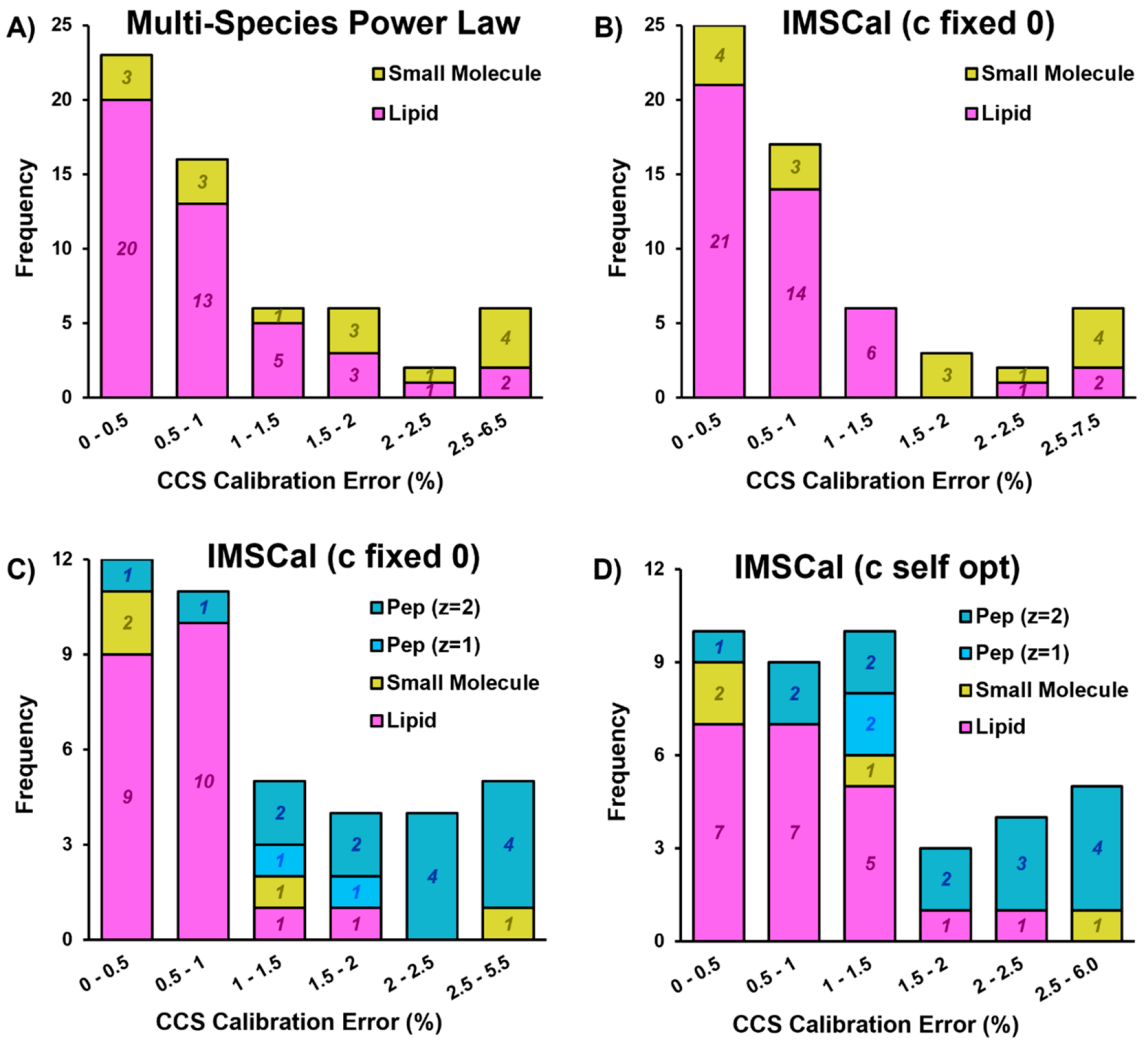
(A) Histogram of NIST SRM lipid and small molecule CCS% errors averaged over 3 days using single power law calibration and (B) IMSCal with “*c*” set to 0. (C) Histogram of NIST SRM lipid and small molecule and enolase/ADH singly and doubly charged peptide CCS% errors calibrated with IMSCal with “*c*” set to 0 and (D) with “*c*” allowed to self-optimize. All calibration parameters can be found in Supporting Information Document 2, Table 9.

Evaluation of calibration errors is insightful but does not represent the performance of the calibration method on analytes that are not contained within the calibration mixture. We applied multispecies power law and IMSCal methods to the same LC and flow injection multiomic data sets presented in Figures 3 and 4. The results, presented in Figure 6, demonstrate that IMSCal performance is comparable to the multispecies power law for small molecules and lipids (Figure 6A,B) based on the number of species with calibration errors below 2%. We have observed for lipids and small molecules that the same features (e.g., choline, valine, cotinine) yield high calibration errors across all calibration methods (see Supporting Information Document 2, Table 10), suggesting that these poorly calibrated features are not likely the result of the calibration methods. When multiply charged peptides are included (Figure 6C,D), the choice to fix IMSCal’s radial term to zero is beneficial to lipids but does not have an impact on its performance for small molecules or singly charged peptides. Self-optimization of the c-term yields one additional doubly charged peptide with a calibration error below 2%. Compared to MOCCal, IMSCal and the multispecies power law provides a similar number of lipid species with errors below 2% (MOCCal = 43; multispecies power law = 41; IMSCal = 41) and identical results for small molecules (10 below 2% for all). When multiply charged peptides are included, MOCCal’s implementation of power law calibration provides errors less than 2% for nearly 2× more peptides than IMSCal (MOCCal = 12; IMSCal (*c* = 0) = 6; IMSCal (*c* self-opt) = 7).

In general, our comparison of calibration methods for generating ^TW^CCS_N2_ demonstrates consistency across single-species power law, multispecies power law, and the IMSCal method. The majority of lipids and small molecules are calibrated within 2% of available ^DT^CCS_N2_ values using any of the three methods. This level of accuracy is sufficient to enable the use of ^TW^CCS_N2_ as a parameter for identification of unknown features within untargeted single or multiomic experiments.

## Conclusions

Although ion mobility mass spectrometry can resolve different types of biomolecules based on their structures, several analytical challenges have limited the utility of IM-MS for simultaneous multiomic analyses of complex biological samples. For traveling wave IM-MS in particular, one of these challenges has been the ability to obtain calibrated CCS values that agree with DTIM measurements, upon which most CCS databases and prediction tools are based. It is well documented that calibrants that are structurally similar to the analyte provide the most accurately calibrated ^TW^CCS_N2_ values. In practice, the application of a single calibration equation to convert arrival times to ^TW^CCS_N2_ values is more convenient when dealing with large data sets or heterogeneous mixtures of analytes. The single-equation approach also alleviates the complication of determining which set of calibrants and CCS calibration curve to use on an unknown feature without first determining its identity. The trade-off for convenience is the introduction of calibration biases that may confound or prohibit the use of calibrated ^TW^CCS_N2_ values in the feature identification process by direct matching or filtering approaches.

To streamline the implementation of structurally matched multiomic TWIM calibrations, we have developed an automated tool, MOCCal, for the assignment of features to a calibrant set/CCS calibration curve without any *a priori* knowledge of the feature’s classification or identity. This process takes advantage of the evidence that structurally matched calibrations provide the most accurate calibrated ^TW^CCS_N2_ values by evaluating the difference, ΔCCS, in Å^2^ between the calibrated ^TW^CCS_N2_ value and the ^DT^CCS_N2_ vs *m*/*z* trendline for the same biochemical class as the calibrants. We demonstrate that the smallest difference in CCS most often occurs when the calibrant matches the analyte and that MOCCal consistently assigns features to the matching class/calibrant using this process. While some lipid and small molecule features may be assigned to calibrations that do not match their chemical class, we have demonstrated that this outcome still provides accurate calibrated ^TW^CCS_N2_ values. The calibration effect scoring system implemented in MOCCal allows users to identify these situations as well as the opposite case of a clear best-fit calibrant.

We have developed MOCCal around small molecule, lipid, and peptide calibrations with the intention of enabling mixed multiomic analyses on TWIM-MS platforms. Within those constraints, users of MOCCal will have the flexibility to determine the composition of the calibrant mixtures that best suit their needs and resources. MOCCal is open-source and freely available online at github.com/HinesLab/MOCCal. Documentation including guides and examples is available as well. User-friendly GUIs have been implemented in MOCCal to allow for easy accessibility by users at any level of programming competency. If raw calibration data have already been processed, the stand-alone MOCCal application can be used for class assignment and CCS calibration. For the raw calibration data itself to be used, DEIMoS is a prerequisite download required for the functionality of MOCCal.^30,37^ User-driven expansion of MOCCal for the calibration of negative ionization mode data sets and the incorporation of other endogenous or xenobiotic chemical classes is welcomed.

## Supporting Information Available

Document 1 containing information on calibrant standards, equations for CCS calculations, zoomed Figure 2, information on the calibration effect parameter, lipid vs peptide calibrations of LysoPCs, and MOCCal performance with ramped TW (PDF)
Document 2 containing CCS Compendium ^DT^CCS_N2_ values, other reference CCS values and sources, MOCCal performance metrics, and all parameters for IMSCal, single power law, and MOCCal calibrations seen in the manuscript (XLSX)
