# Supplementary material for: MOCCal: A Multiomic CCS Calibrator for Traveling Wave Ion Mobility Mass Spectrometry
Source: Anal Chem. 2024 Jan 9;96(3):1185–94. doi: 10.1021/acs.analchem.3c04290 (PMC10809277; doi:10.1021/acs.analchem.3c04290)
Supplement: Supplementary file 1 — ac3c04290_si_001.pdf [file ac3c04290_si_001.pdf]

Supporting Information for

MOCCal: A Multi-Omic CCS Calibrator for Traveling Wave Ion Mobility Mass Spectrometry

Hannah M. Hynds[a] and Kelly M. Hines[a]*

[a]*Department of Chemistry, University of Georgia, Athens, Georgia 30602, United States*

*Address reprint requests to Kelly M. Hines: Department of Chemistry, University of Georgia, 302 East Campus Road, Athens, GA 30602; 706-542-1966; kelly.hines@uga.edu

Table of Contents

1. Lipids……………………………..………………………………………………………….……………………................. S3
2. Calibration Solution Information...…………………..………...…………………………………………….…………….… S3
3. Calibration Curve Calculations.....………………….…………..……………………………………………….…………….… S4
4. Calibrated CCS Calculation.……………………………………………………………………………………………...S4
5. Zoomed Versions of Manuscript Figure 2.……………………………….……………………...……………………...S5
6. Calibration Effect Calculation and Scores……………………………………………………………………………S6
7. Calibration Effect Examples..……………………………………………………………………………….............S7
8. Lipid vs. Peptide Calibration of LysoPCs…………………………………………………………………………….S8
9. MOCCal Performance with Ramped TW……………………………………………………………………......S9

1. Lipids

All lipids below were acquired from Avanti Polar Lipids in Alabaster, Alabama.

Table S1. PC and PE lipid standards used in manuscript.

Lipid	Avanti Product Number	Exact Mass	CAS Number
**PC 10:0/10:0**	850325	565.37	3436-44-0
**PC 12:0/12:0**	850335	621.437	18194-25-7
**PC 14:0/14:0**	850345	677.5	18194-24-6
**PC 16:1/16:1**	850358	729.531	4724-96-3
**PC 18:1/14:0**	850474	731.547	95896-56-3
**PE 10:0**	850700	523.327	253685-27-7
**PE 12:0**	850702	579.39	59752-57-7
**PE 14:0**	850745	635.453	998-07-2
**PE 16:1/16:1**	850706	687.484	61599-23-3
**PE 16:0**	850705	691.515	923-61-5

2. Calibration Solution Information

Table S2. Lipid, Metabolite, and Peptide standard concentrations used in manuscript.

Standard	Concentration (μM)
**PC 10:0**	0.1
**PC 12:0**	0.2
**PC 14:0**	0.5
**PC 16:1**	0.5
**PC 18:1-14:0**	0.5
**PE 10:0**	0.2
**PE 12:0**	0.5
**PE 14:0**	0.5
**PE 16:1**	1
**PE 16:0**	1
**Acetaminophen**	6
**L-Histidine**	2.5
**Caffeine**	3
**Carnosine**	3
**AMP**	4
**Sucrose**	3
**SAM**	5
**Poly-DL-Alanine**	12.67 μg/mL

3. Calibration Curve Calculations

Eqn 1. Correction of arrival times for mass dependent flight time, where t'_a is the corrected arrival time, t_a is the arrival time of the calibrant ion, *m/z* is mass-to-charge of the calibrant ion, and EDC is the enhanced duty cycle delay coefficient. EDC was equal to 1.55 for the instrument used in this work but will vary between instruments.

$$t'_a = t_a - \left(\frac{\left(\sqrt{m/z} * EDC\right)}{1000}\right)$$

Eqn 2. Reduced mass formula where μ is the reduced mass, *m* is the mass of the calibrant, and 28.0134 is the mass of nitrogen.

$$\mu = \frac{m * 28.0134}{m + 28.0134}$$

Eqn 3. Corrected CCS formula where Ω' is the corrected CCS, $^{DT}CCS_{N2}$ is the literature CCS value obtained using drift-tube ion mobility, *z* is the charge of the calibrant, and μ is the reduced mass. The corrected CCS can be expressed in a relationship to the corrected arrival time t'_a through power law regression. *A'*, t_0, and *B* are the regression's optimized parameters.

$$\Omega' = \frac{^{DT}CCS_{N2} * \sqrt{\mu}}{z} = \frac{A' * (t'_a + t_0)^B}{z}$$

4. Calibrated CCS Calculation

Eqn 4. CCS calibration formula where Ω is a feature's calibrated CCS, t'_a is corrected arrival time, *z* is the charge, μ is the reduced mass, and *A'*, t_0, and *B* are parameters optimized through power law regression.

$$\Omega = \left(\frac{A'}{\sqrt{\mu}} * (t'_a + t_0)^B\right) * z$$

5. Zoomed Versions of Manuscript Figure 2

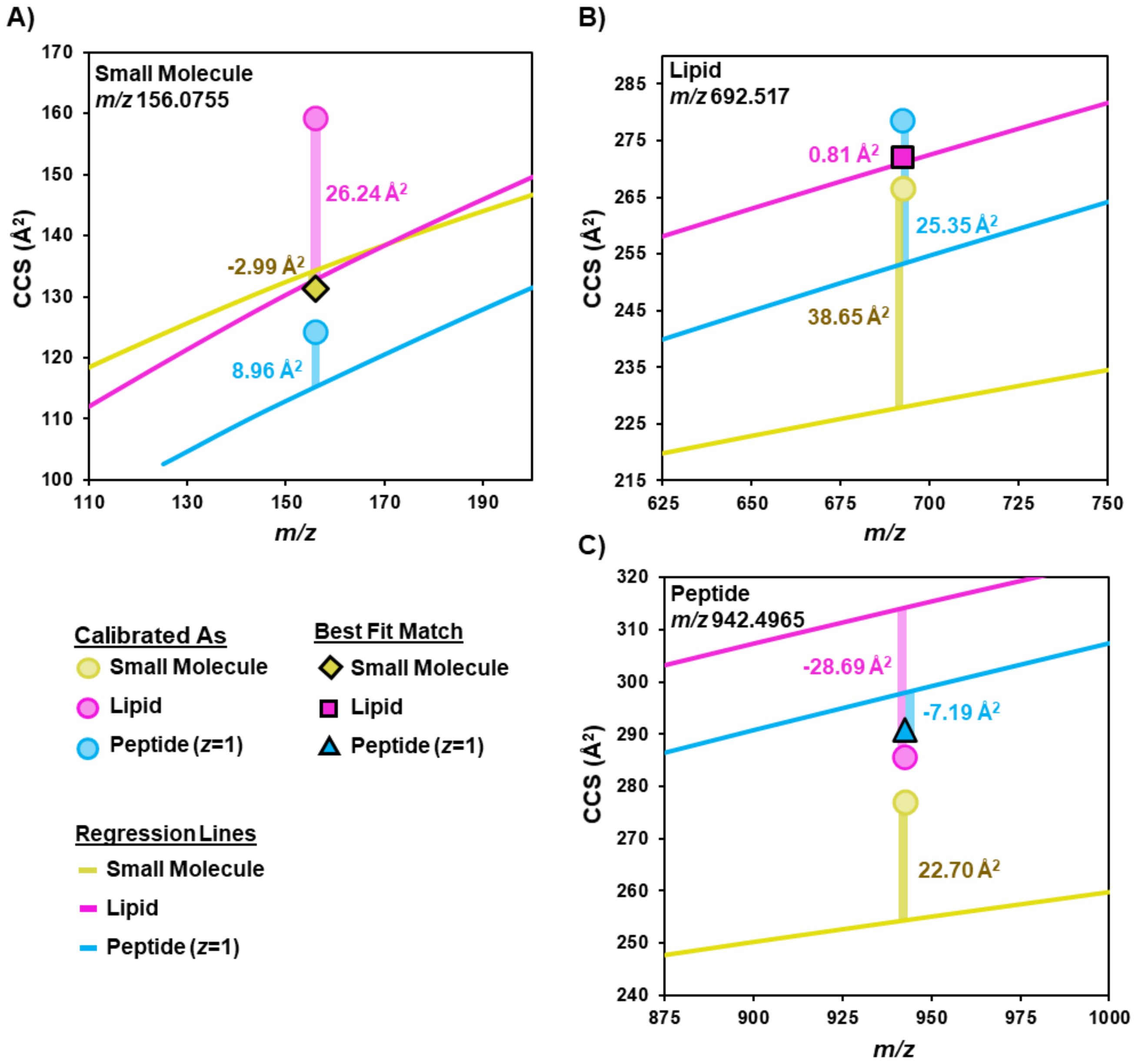

Figure S1. Zoomed panels for the **A)** small molecule **B)** lipid and **C)** peptide features seen in manuscript Figure 2 to illustrate the class assignment process.

For each feature, its *m/z* value is used to obtain small molecule, lipid, and singly-charged peptide CCS values according to their respective trendlines. Additionally, its *m/z* and arrival time values are used to obtain class-specific calibrated CCS values for the three classes as well. The difference between the class-specific pair of trendline and calibrated CCS values are used for class assignment. **Figure S1A** shows a small molecule feature, **Figure S1B** shows a lipid feature, and **Figure S1C** shows a singly-charged peptide feature.

6. Calibration Effect Calculation and Scores

Eqn 5. CCS Error equation where CCS_1 is the value calculated using the calibration curve of the assigned class and CCS_2 is the next closest CCS value to that of the assigned class. The results of this will indicate the "Calibration Effect" seen in **Table S3**

$$CCS\ Error = \left(\frac{|CCS_1 - CCS_2|}{CCS_1}\right) * 100$$

Table S3. The Calibration Effect resulting from the CCS Error (**Eqn 5)** between the assigned class' calculated CCS value and the next closest value.

Calibration Effect	CCS Error
**4**	X > 6
**3**	3 < X <= 6
**2**	1 < X <= 3
**1**	X <= 1
**0**	MULTIPLY CHARGED

7. Calibration Effect Examples

Table S4. Interpretations of different calibration effect scores.

Score	Compound	Class Assignment	Class Assigned CCS	Peptide (z=1) CCS	CCS Error
**1**	Sucrose	Small Molecule	173.176	171.799	0.80
**2**	PC 36:2	Lipid	291.162	295.859	1.61
**3**	Caffeine	Small Molecule	140.503	134.784	4.07
**4**	Choline	Small Molecule	123.189	114.548	7.01

The calibration effect score portrays the extent to which calibrating with a certain class's calibration curve affects the final resulting CCS value. For example, in the cases above, the closest CCS value to that of the assigned class is the value that results from using the singly-charged peptide calibration curve. For sucrose, the calibration effect score is 1. This is because the resulting CCS value when using the small molecule calibration curve yields an extremely similar result to the resulting CCS value from the peptide curve. As the score goes up, the more significant difference there is between the results yielded from different class's calibration curves. By the time the score gets to 4, as with choline above, the results from different calibration curves could be significantly different.

8. Lipid vs. Peptide Calibration of LysoPCs

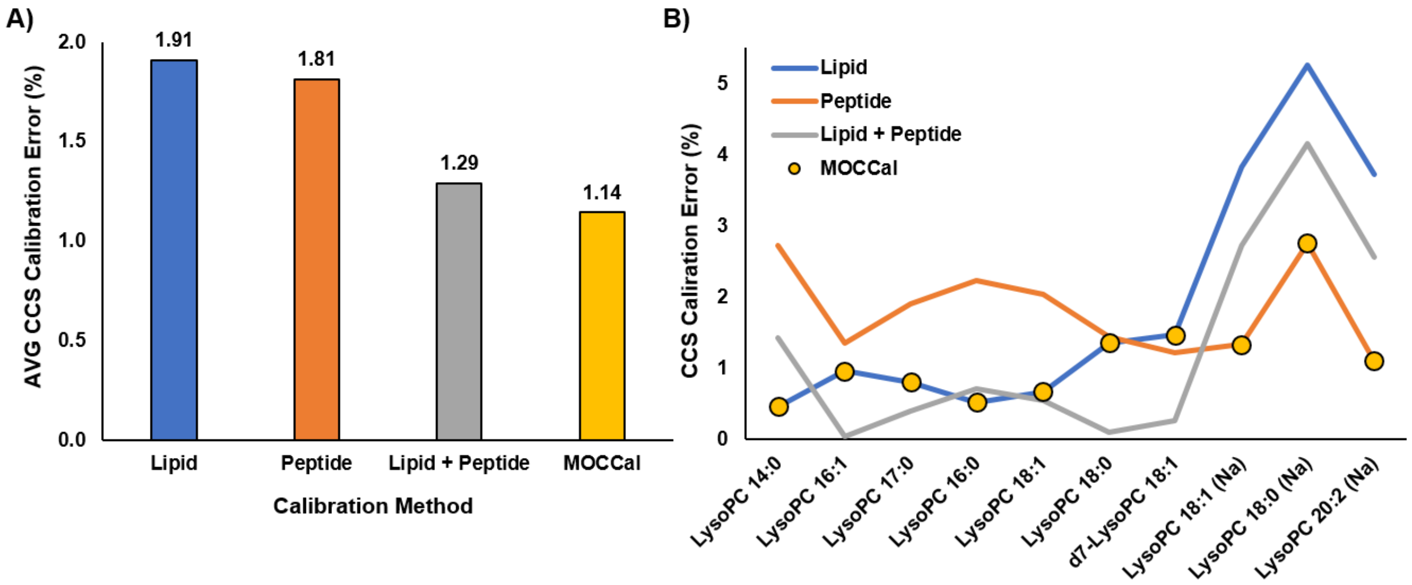

Figure S2. A) Bar chart of NIST SRM LysoPC lipid average CCS calibration errors (%) and **B)** line chart of individual NIST SRM LysoPC lipid CCS calibration errors (%) when calibrated with the lipid calibration curve (blue), peptide calibration curve (orange), single power law curve built with lipids and peptides (gray), and MOCCal (yellow). As seen in panel B, MOCCal selected 7 of the LysoPCs to be calibrated using the lipid calibration curve, with the remaining 3 calibrated using the peptide calibration curve. As seen in panel A, this selection resulted in a lower average CCS calibration error than a single power law calibration curve or either class-specific calibration curve alone.

9. MOCCal Performance with Ramped TW

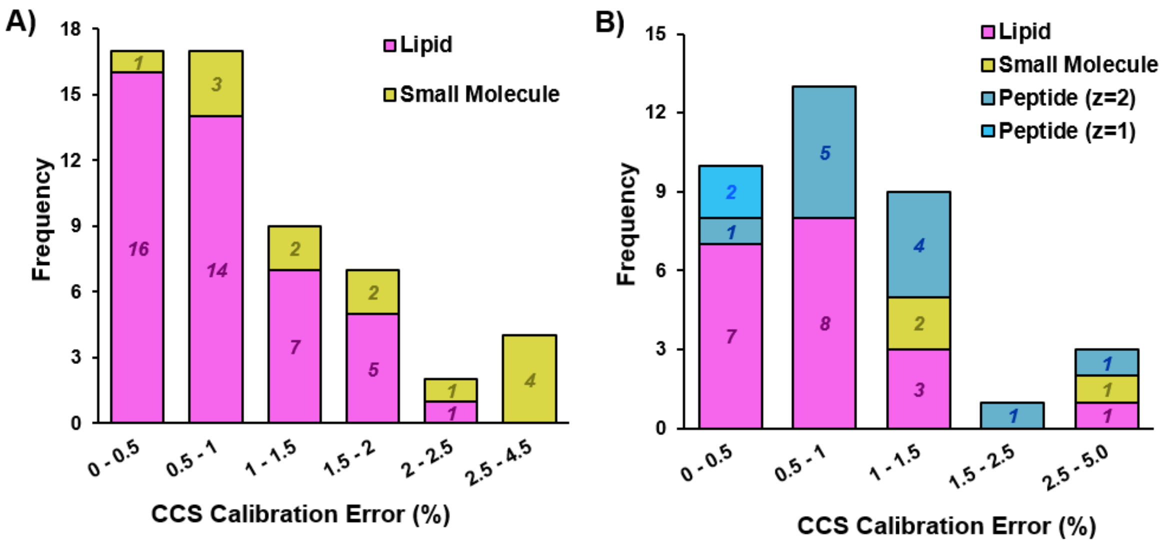

Figure S3. A) Histogram of NIST SRM lipid and small molecule CCS % errors and **B)** NIST SRM lipid and small molecule and enolase/ADH singly and doubly-charged peptide CCS % errors using a ramped IM wave velocity of 600-200 m/s instead of a fixed velocity of 600 as seen in the other manuscript figures.
